# 2085. PrEP Interest and Preferences Among US Black and Hispanic Men – A National Survey

**DOI:** 10.1093/ofid/ofac492.1707

**Published:** 2022-12-15

**Authors:** Tonia Poteat, Supriya Sarkar, Leigh Ragone, Keith Rawlings, Alex R Rinehart, Jennifer N Hill, Kyli Gallington, Karin S Coyne, Vani Vannappagari

**Affiliations:** University of North Carolina School of Medicine, Durham, North Carolina; ViiV Healthcare, Baltimore, Maryland; ViiV Healthcare, Baltimore, Maryland; ViiV Healthcare, Baltimore, Maryland; ViiV Healthcare, Baltimore, Maryland; Evidera, Bethesda, Maryland; Evidera, Bethesda, Maryland; Evidera, Bethesda, Maryland; ViiV Healthcare, Baltimore, Maryland

## Abstract

**Background:**

Use of daily oral HIV pre-exposure prophylaxis (DO PrEP) has increased steadily in the past several years, but patterns of racial disparities have emerged in PrEP uptake. Although Black and Hispanic people are disproportionately affected by HIV in the US, they constitute a minority among those accessing DO PrEP. Newly available prevention options, such as long-acting injectable (LA) PrEP may help close the gap in unmet need for PrEP; however, interest in LA PrEP has not been evaluated specifically among racial/ethnic minority groups. Awareness, willingness, and usage of PrEP as well as HIV prevention preferences were assessed among sexually active adult men in the US.

**Methods:**

Participants were recruited through a geographically targeted social media campaign and completed a self-administered, cross-sectional, online survey on demographics, sexual health and behavior, healthcare access, PrEP awareness and usage, and PrEP intention and preferences. Eligible participants met the following criteria: cisgender men, self-identified Black race and/or Hispanic ethnicity, 18 years or older, reporting unknown or HIV-negative status, currently residing in the US, and reporting anal or vaginal sex in the past six months. Descriptive statistics were calculated using SAS v9.4.

**Results:**

From November to December 2021, 1365 men completed the survey (median age: 29.0 years; Black non-Hispanic: 43.1%, Black Hispanic: 40.3%, White Hispanic: 10.2%, Other Hispanic: 6.4%). A majority had heard of DO PrEP (66.6%) and LA PrEP (47.5%) as a way to prevent HIV; however, a smaller proportion had spoken to a healthcare provider (HCP) about PrEP (42.1%), had ever used PrEP (24.7%), or were currently taking PrEP (16.3%) (Figure 1). A large majority (74.0%) reported interest in using LA PrEP. When asked about their preferred PrEP option, 60.2% chose LA PrEP and 7.4% chose DO PrEP, while 27.5% stated that they preferred neither PrEP option.

**Figure 1.**
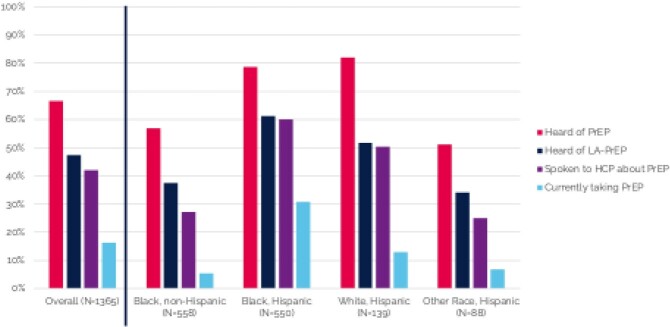
PrEP Awareness, Discussion with HCP, and Usage Among US Black and Hispanic Men, Overall and by Race/Ethnicity

**Conclusion:**

Most participants demonstrated high awareness of PrEP and a strong interest in LA PrEP. The availability of and interest in LA PrEP may serve as an opportunity to help increase overall PrEP uptake among Black and Hispanic men in the US.

**Disclosures:**

**TONIA POTEAT, PhD, MPH, PA-C**, ViiV Healthcare: Advisor/Consultant **Supriya Sarkar, PhD, MPH**, ViiV Healthcare: Salary|ViiV Healthcare: Stocks/Bonds **Leigh Ragone, MS**, GlaxoSmithKline: Stocks/Bonds|ViiV Healthcare: Employment **Keith Rawlings, MD**, ViiV Healthcare: Employee **Alex R. Rinehart, PhD**, ViiV Healthcare: Stocks/Bonds **Vani Vannappagari, MBBS, MPH, PhD**, ViiV Healthcare: I am full time employee of ViiV Healthcare and receive GlaxoSmithKline stock as part of my compensation package|ViiV Healthcare: Stocks/Bonds.

